# A Look Into the Power of fNIRS Signals by Using the Welch Power Spectral Estimate for Deception Detection

**DOI:** 10.3389/fnhum.2020.606238

**Published:** 2021-01-18

**Authors:** Jiang Zhang, Jingyue Zhang, Houhua Ren, Qihong Liu, Zhengcong Du, Lan Wu, Liyang Sai, Zhen Yuan, Site Mo, Xiaohong Lin

**Affiliations:** ^1^College of Electrical Engineering, Sichuan University, Chengdu, China; ^2^China Mobile (Chengdu) Industrial Research Institute, Chengdu, China; ^3^College of Biomedical Engineering, Sichuan University, Chengdu, China; ^4^School of Information Science and Technology, Xichang University, Xichang, China; ^5^Sichuan Cancer Hospital and Institute, Chengdu, China; ^6^Institutes of Psychological Sciences, Hangzhou Normal University, Hangzhou, China; ^7^Department of Psychology, Zhejiang Normal University, Jinhua, China; ^8^Bioimaging Core, Faculty of Health Sciences, University of Macau, Taipa, China

**Keywords:** functional near-infrared spectroscopy, power, Welch power spectrum estimation, deception, quantitative analysis

## Abstract

Neuroimaging technologies have improved our understanding of deception and also exhibit their potential in revealing the origins of its neural mechanism. In this study, a quantitative power analysis method that uses the Welch power spectrum estimation of functional near-infrared spectroscopy (fNIRS) signals was proposed to examine the brain activation difference between the spontaneous deceptive behavior and controlled behavior. The power value produced by the model was applied to quantify the activity energy of brain regions, which can serve as a neuromarker for deception detection. Interestingly, the power analysis results generated from the Welch spectrum estimation method demonstrated that the spontaneous deceptive behavior elicited significantly higher power than that from the controlled behavior in the prefrontal cortex. Meanwhile, the power findings also showed significant difference between the spontaneous deceptive behavior and controlled behavior, indicating that the reward system was only involved in the deception. The proposed power analysis method for processing fNIRS data provides us an additional insight to understand the cognitive mechanism of deception.

## Introduction

Deception is a universally existing sociopsychological phenomenon, involving such psychological activities as perception, memory, thinking, and imagination (Depaulo et al., 2003; Crossman and Lewis, 2006; Talwar and Kang, 2008; Kang, 2013). To date, great efforts have been made to reveal the neurobiological basis of deception. For example, electroencephalography (EEG)/event-related potential (ERP) (Fukuda, 2001; Carrión et al., 2010), functional magnetic resonance imaging (fMRI) (Harada et al., 2009; Abe, 2011; Gamer et al., 2012), functional near-infrared spectroscopy (fNIRS) (Bhutta et al., 2015; Zhang et al., 2016), and various data analysis methods (Christ et al., 2009; Zhang et al., 2016) were used to elicit the neural mechanisms underlying deception. Nevertheless, the neural mechanisms for deception still remains unclear, and the related data analysis methods need to be developed.

Because of poor EEG/ERP inherent spatial resolution, researchers are not better enabled to know about the brain regions involved in deception until the advent of fMRI (Fukuda, 2001; Harada et al., 2009; Carrión et al., 2010; Abe, 2011). Compared to fMRI, fNIRS can be operated in a portable, comfortable, and quiet way with fewer body constraints (Beurskens et al., 2014). fNIRS that relies on the hemodynamic responses to infer brain activation has also been extensively utilized to inspect the cognition and brain disorders (Hoshi, 2009; Izzetoglu et al., 2007; Caliandro et al., 2013; Yuan, 2013; Ding et al., 2014; Ren et al., 2019). In particular, fNIRS as an optical neuroimaging tool can provide the quantitative hemodynamic information including oxyhemoglobin (HbO) and deoxyhemoglobin concentration changes, which plays an important role in the study of cognitive processing in the frontal/prefrontal cortex (PFC) (Izzetoglu et al., 2007; Hoshi, 2009; Caliandro et al., 2013; Yuan, 2013; Ding et al., 2014; Ren et al., 2019). More importantly, fNIRS has exhibited its unbeatable advantages in deception detection that involves the inspection of executive functions in PFC including withholding the truth and response monitoring (Hoshi, 2003; Kovelman et al., 2008; Ayaz et al., 2012). Despite all these, deception still remains a profound paradigm for studying human behaviors, in view of the great complexity of deception in different environments and thus the limited understanding of neural mechanisms underlying various situations.

In view of the fact that the neural activities are accompanied by energy and power variation, the power changes of hemodynamic response, capable of reflecting brain activity intensity (Buxton et al., 2004; Zhang et al., 2010b), this study proposes a power analysis method based on the Welch power spectrum estimation of fNIRS signals including hemodynamic information, in a bid to quantify the brain hemodynamic responses associated with deception underlying various behavior states. The Welch power spectrum algorithm, as an effective spectral estimation method without seriously destroying the resolution, can significantly reduce the variance of spectral estimation by segmenting data through overlapping and adding windows in time domain (Welch, 1967; Proakis et al., 2003). We have made extensive efforts in acquiring data, documents, or information regarding the application of power analysis of fNIRS in exploring brain functional activity, which turns out to be a very rarely adopted practice, with little references available. But we do find the power measure produced by the power analysis model capable of quantifying the brain activity energy in different brain regions during a short-time period, and it has been applied in fMRI and EEG (Zhang et al., 2010a; Radulescu et al., 2012; Gonzalez et al., 2016). And this leads us to assume that the power of fNIRS may also be able to serve as a neuromarker to unveil the brain power difference between the spontaneous deceptive and controlled behavior. In short, the novel measure in fNIRS proposed in this study is rather reasonably expected to be a valuable new approach to better understanding the cognitive mechanism associated with deception.

## Materials and Methods

### Participants

Twenty-five participants (14 females and 11 males; all aged 19–22 years) were recruited for the fNIRS experiments. All participants were right handed, who had no reported histories of brain diseases. Participants were instructed to sign informed consent documents prior to data acquisition. The protocol of this study was approved by the ethics committees of the Zhejiang Normal University, and the experimental tests were performed in accordance with the guidelines.

### Tasks and Procedures

**FIGURE 1:**
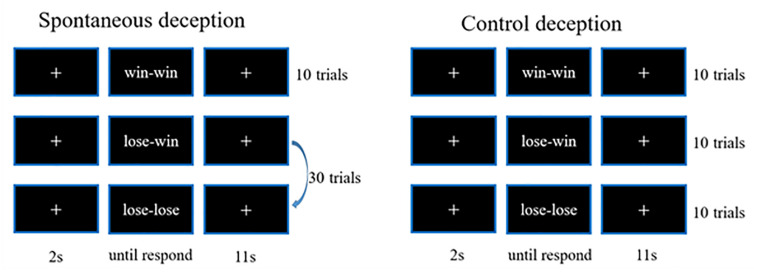
The schematic of paradigm for deception tests.

The paradigm adopted for the present study consisted of two blocks: one for spontaneous deceptive behavior and the other for controlled behavior. The spontaneous deceptive behavior block contained 40 event-related trials, whereas the controlled behavior block had 30 trials. The stimuli ended as soon as participants respond, and each trial included a 2 s prestimuli period, followed by an 11 s post-stimulus and recovery period with a white fixation cross displayed in the center of the monitor screen to make sure the hemodynamic response returned to the baseline (Figure 1).

During the stimuli period of each trial, participants were instructed to play a computer poker game with an opponent in a separated room. The winner was the poker game player who scored more points in each round (trial). For the spontaneous deceptive behavior stimuli, only the opponent picked up the first card of the poker game, and then only the participant who was able to see both his (her) and the opponent’s cards needed sent the final results of the competition (answers) to the opponent by pressing a button. The winner (the participant or the opponent) should be rewarded some amount of money. Interestingly, if the participant won the game for each round, he (she) generally sent the correct answer (win–win) to the opponent, whereas when the participant lost the game, he (she) might send a false answer to the opponent (lose–win) to receive a reward by deception or send the correct answer to lose the game (lose–lose). Among the 40-trial spontaneous deceptive behavior stimuli, 10 were for the win case, 30 for the lose case. Participants did not need to lie in the win case, whereas participants could lie for more money in the lose case. Therefore, 30 trials were designed for lose case to maintain the trials for lie (lose–win) or truth telling (lose–lose) were enough (about 10) for calculation.

By contrast, for the controlled behavior stimuli, participants needed to follow instructions on the computer screen to tell the truth or lie. In addition, the winner was not awarded any money for the controlled behavior task. The controlled behavior task consisted of three conditions: participants won the poker game and then sent the right answer (win–win) to the computer, and participants lost the game and sent the false (lose–win) or the true answer (lose–lose) to the computer. Ten trial tests were respectively, performed for each of the three conditions (i.e., win–win, lose–win, and lose–lose). And the test cases of spontaneous and controlled behaviors were provided in Table 1. Participants received rewards after they finished the whole experiment.

**TABLE 1:** Test cases of the spontaneous and controlled behaviors.

Categories	Spontaneous behavior (S)			Controlled behavior (C)		
Cases	a	b	c	a	b	c
Real answer	Win	Lose	Lose	Win	Lose	Lose
Participant’ answer	Win	Win	Lose	Win	Win	Lose
Results	Truth	Deception	Truth	Truth	Deception	Truth

### Data Acquisition and Preprocessing

**FIGURE 2:**
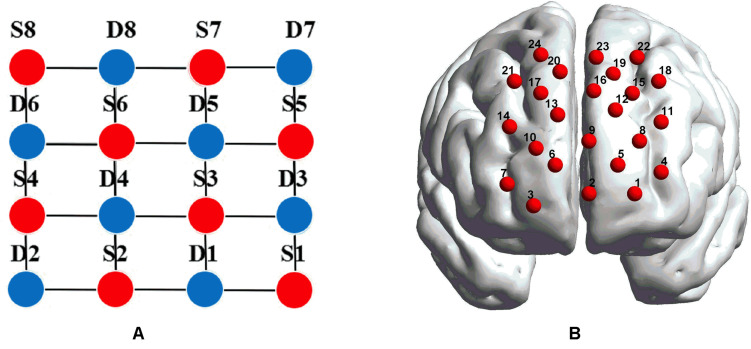
**(A)** The distribution of laser sources and optical detector pair. The red dots represent the laser sources, whereas the light detectors are denoted by the blue dots, and the gray lines define the generated 24 channels. **(B)** The placements of the 24 channels in the dorsal bilateral frontal region. **(B)** was obtained by importing the 3D coordinates measured by a 3D digitizer into BrainNet Viewer to generate the configuration of 24 channels.

fNIRS recordings were performed with a CW fNIRS system (ETG-4000, Hitachi Medical Co., Japan; 24 channels with eight laser sources and eight optical detectors). The optodes were placed on a 9 × 9 cm patch that was able to cover the frontal lobe (Figures 2A,B). The distance between each source and each detector was 30 mm; the sampling rate of the ETG-4000 system was 10 Hz, and the wavelengths of laser sources used were 695 and 830 nm. A three-dimensional (3D) magnetic space digitizer (EZT-DM401, Hitachi Medical Corporation, Japan) was utilized to measure the 3D spatial location of each optode on each participant’s scalp. And then the NIRS-SPM software (Ye et al., 2009) was used to access each channel’s mean MNI standard coordinates (Singh et al., 2005), which are provided in Table A1.

The fNIRS data were first processed by using a 0.01 Hz temporal high-pass filter and subsequently 0.3 Hz low-pass filter to remove baseline drifts and pulsation due to the heartbeat (Ding et al., 2014; Sai et al., 2014). In this study, only HbO signals were processed for further power analysis because of its high signal-to-noise ratio (Homae et al., 2007; Lu et al., 2010; Ding et al., 2014). Two participants were excluded from further analysis because of the poor quality of their optical data (possibly due to poor contact between the optodes and the scalp). Next, the datasets were segmented in relation to different markers that included three types of triggers for the spontaneous behaviors and three additional categories of triggers for the control behaviors (i.e., the win–win, lose–win, and lose–lose behaviors).

### Data Analysis

For the trial-averaged HbO data from all channels, the PWELCH function in MATLAB is first used to calculate the Welch power spectral density (PSD) (Welch, 1967; Proakis et al., 2003) for each time series relevant to each channel and each participant. Interestingly, previous studies (Efron and Tibshirani, 1993) showed that the Welch algorithm can break down the original signal into *L* overlapped segments. Consequently, the Welch power spectrum estimation algorithm of HbO signal *x*(*n*) is defined as follows:

1) The signal *x*(*n*) with length *N* is divided into *L* data segments with length *M*, and the data segment is allowed to overlap, and *N* is the number of data points for each trial. The Welch power spectrum of *x*_*i*_(*n*), data segment *i*, is denoted as *P*_*i*_(*w*) (Proakis et al., 2003),
  (1) $$P_{i}\left(w\right)=\frac{1}{MU}{\left|\sum_{n=0}^{M-1}x_{i}\left(n\right)d_{2}\left(n\right)e^{-jwn}\right|}^{2}$$

in which $U=\frac{1}{M}\sum_{n=0}^{M-1}d_{2}^{2}\left(n\right)$ is the normalized factor that ensures that the obtained power spectrum is asymptotically unbiased estimation; *d*_2_(*n*) is the window function, and *w* is the angular frequency.

2) After calculating the Welch power spectrum of each data segment, we then generate the mean power spectrum of all *L* segments and obtain the Welch power spectrum estimation of the whole HbO signal *x*(*n*) (Proakis et al., 2003),
  (2) $$p\left(w\right)=\frac{1}{L}\sum_{i=1}^{L}P_{i}\left(w\right)=\frac{1}{MUL}\sum_{i=1}^{L}{\left|\sum_{n=0}^{M-1}x_{i}\left(n\right)d_{2}\left(n\right)e^{-jwn}\right|}^{2}$$
3) Once the Welch power spectrum estimation of the *m*th channel for *k*th participant *p*_*m*,*k*_(*w*) is determined, the power is generated, which is the sum of PSD from all angular frequencies,
  (3) $$P_{m,k}=\sum_{w}p_{m,k}\left(w\right)$$

in which *P*_*m,k*_ is the power of the *m*th channel from the *k*th participant.

In addition, the standardized indicators for power are used to eliminate the effects of individual differences, in which *P*_*m,k*_ is standardized,

(4) $$\text{Standard}.P_{m,k}=P_{m,k}/\text{mean}\left(P_{k}\right)$$

where Standard.*P*_*m*,*k*_ is the standardized form of *P*_*m,k*_, *P*_*k*_ denotes the data vector with the total power from 24 channels for the *k*th subject, and mean(*P*_*k*_) is the mean value of the data vector *P*_*k*_.

As a result, a single power was produced for each channel from each participant based on Eqs 1–3, and then the power was standardized with Eq. 4. Further statistical analysis was performed by using the standardized indicator measures to identify the channels that exhibited significant difference between various conditions (Efron and Tibshirani, 1993; Nichols and Holmes, 2001; Manly, 2006; Zhang et al., 2010a, 2011).

## Results and Discussion

The Welch power spectrum estimation of HbO signals was first generated for each channel from each subject. Although the power spectrum at characteristic points may be specified as the index of brain functional activation, brain activity may affect the power of signals at multiple frequencies. So we calculated the changes of average power for fNIRS signals in time domain. In order to obtain the average power in time domain by power spectrum, it is necessary to calculate the power of signals within all frequency bands. And the power as a novel neuromarker was produced for each channel from each subject, which was utilized as a quantitative measure for the following statistical analysis.

**FIGURE 3:**
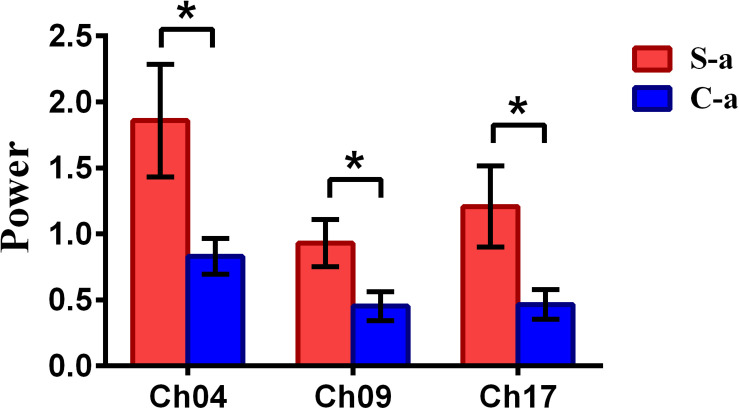
The channels with statistically significant difference in the power (mean ± SE) between the spontaneous deceptive and controlled behavior for the win–win case. The red and blue colors denote the spontaneous deceptive and controlled behavior, respectively. The horizontal axes denote the channels with statistically significant differences, and the vertical axes denotes the power (mean ± SE) of the channels. *p < 0.05 (the *p*-values are from *t*-test to show the difference between the spontaneous deceptive and controlled behavior for the win–win condition.) Here S-a represents the win–win case under the conditions of spontaneous behavior (case of spontaneous behavior in Table 1). C-a represents the win–win case under the conditions of control behavior.

**FIGURE 4:**
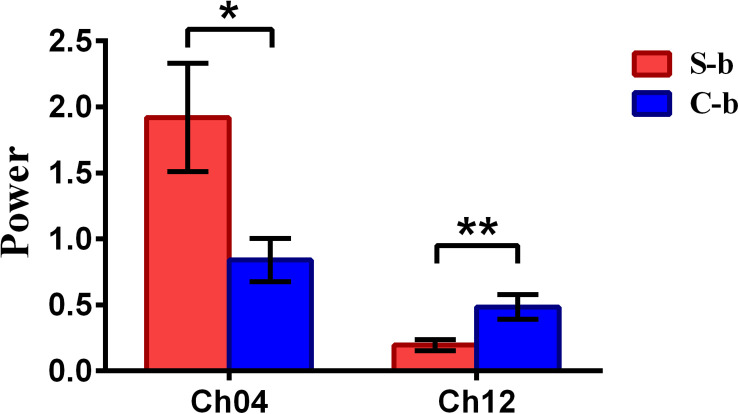
The channels with statistically significant difference in the power (mean ± SE) between the spontaneous deceptive and controlled behavior for the lose–win case. The red and blue colors denote the spontaneous deceptive and controlled behavior, respectively. The horizontal axes denote the channels with statistically significant differences, and the vertical axes denote the power (mean ± SE) of the channels. *p < 0.05 and **p < 0.01. (The *p*-values are from *t*-test to show the difference between the spontaneous deceptive and controlled behavior for the lose–win condition.) Here S-b represents the lose–win case under the conditions of spontaneous behavior. C-b represents the lose–win case under the conditions of control behavior.

**FIGURE 5:**
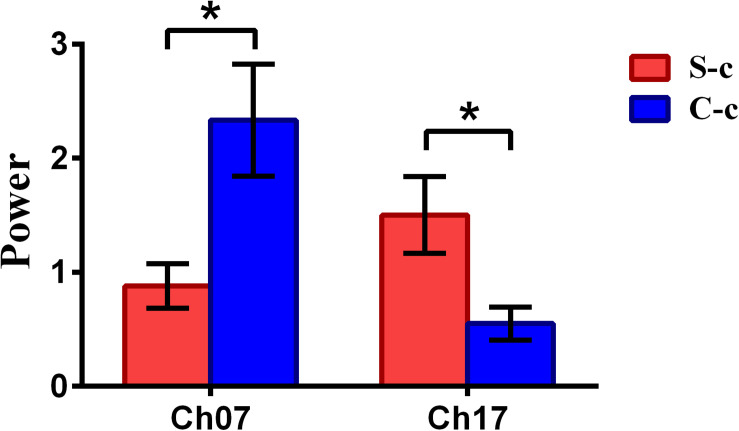
The channels with statistically significant difference in the power (mean ± SE) between the spontaneous deceptive and controlled behavior for the lose–lose case. The red and blue colors denote the spontaneous deceptive and controlled behavior, respectively. The horizontal axes denote the channels with statistically significant differences, and the vertical axes denote the power (mean ± SE) of the channels. *p < 0.05. (The *p*-values are from *t*-test to show the difference between the spontaneous deceptive and controlled behavior for the lose–win condition.) Here S-c represents the lose–lose case under the conditions of spontaneous behavior. And C-c represents the lose–lose case under the conditions of control behavior.

To inspect the neural correlates of deception, the paired *t* statistical test based on power measure was performed to examine the brain power differences between the spontaneous and controlled behaviors for the win–win, lose–win, and lose–lose cases. Interestingly, it was discovered from Figure 3 that for the win–win case, the spontaneous behavior exhibited significantly larger power than that from the controlled behavior in the frontopolar area (BA10, channel 04, and channel 09) and dorsolateral PFC (DLPFC) (BA09, channel 17). Meanwhile, the results in Figure 4 showed that for the lose–win case, the spontaneous deceptive behavior exhibited significantly higher power than the controlled behavior in the frontopolar area (BA10, channel 04), whereas the power for the controlled behavior was significantly enhanced as compared to that from the spontaneous behavior in the DLPFC (BA09, channel 12). In addition, we discovered that for the lose–lose case, the brain power for the controlled behavior was significantly higher in the frontopolar area (BA10, channel 07) than that from the spontaneous behavior (Figure 5). However, this is not the case for the brain power in DLPFC (BA09, channel 17), in which the spontaneous behavior exhibited higher value as compared to the controlled behavior.

**FIGURE 6:**
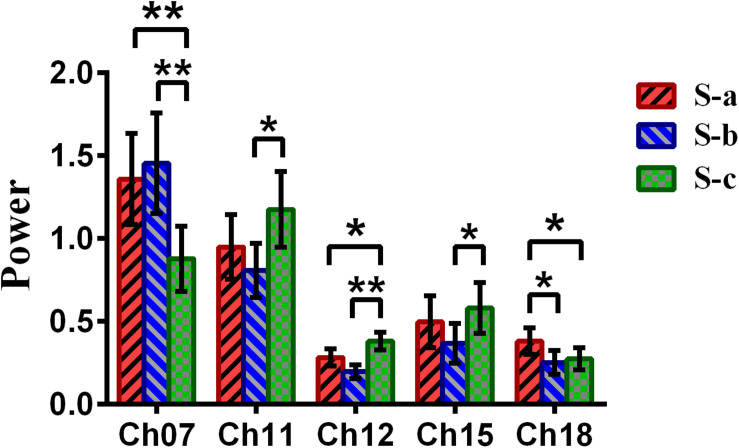
The channels with statistically significant differences in the power (mean ± SE) between the win–win, lose–win, and lose–lose cases underlying spontaneous deceptive behavior. The red, blue, and green colors represent the win–win, lose–win, and lose–lose condition, respectively. The horizontal axes denote the channels with statistically significant differences, and the vertical axes denote the power (mean ± SE) of the channels. *p < 0.05 and **p < 0.01. (The *p*-values are from the repeated-measures analysis of variance measure for the win–win, lose–win, and lose–lose cases underlying the spontaneous deceptive behavior.)

Repeated-measures analysis of variance (ANOVA) measure was performed to explore the difference between the win–win, lose–win, and lose–lose conditions for the spontaneous deceptive behavior. The results in Figure 6 demonstrated that the power between the three cases exhibited significant difference in several brain regions, although this is not the case for the controlled behavior. For example, *post hoc* analysis for the spontaneous behavior showed that for the win–win case, the brain activation in DLPFC (BA09, channel 18) was significantly increased as compared to that from the lose–win case. In addition, compared with the lose–lose case, the lose–win case showed enhanced brain activation in the frontopolar area (BA10, channel 07). Meanwhile, the lose–lose case also exhibited significantly higher power than the lose–win case in the DLPFC (BA46, channel 11; BA09, channels 12 and 15). In particular, compared with the lose–lose case, the win–win case showed significantly higher brain power in the frontopolar area (BA10, channel 07) and DLPFC (BA09, channel 18). Further, the lose–lose case as well manifested significantly higher power than the win–win case in the DLPFC (BA09, channel 12).

In this study, fNIRS was used to inspect the neural mechanism of deception by using the measure of power, built on the basis of the Welch power spectrum estimation. The analysis of signals was not conducted directly based on the changes of signal amplitude at a certain instantaneous time point, but on quantitative comparison and analysis of the changes of power within all frequency bands between different cases. The analysis results of the power within all bands may not be consistent with the results of the amplitude variation of the fNIRS signal at a certain instantaneous time point, due to the fact that the amplitude at a single instantaneous time point is more susceptible to noise. Statistical analysis including the paired *t* test and the ANOVA measure was performed to examine the brain power difference between the spontaneous and controlled behaviors. The power spectrum approach has turned out to be able to detect neural activities for the whole brain using detection tools such as fMRI and EEG (Marchini and Ripley, 2000; Moritz et al., 2003; Duff et al., 2008; Gonzalez et al., 2016). But ours is the first ever study, to the best of our knowledge, that combines the power analysis model and fNIRS data to quantitatively examine the brain functional activation with deception. We discovered that the spontaneous deceptive behaviors exhibited significantly higher power than the controlled behavior. The analysis results also demonstrated that the power can be an effective neuromarker to reveal the complex neural mechanism associated with deception. Interestingly, the identified brain regions such as the right DLPFC (BA09), the left DLPFC (BA46), and the frontopolar area (BA10) are involved in the planning of complex and coordinated movements (Baker et al., 1996; Hoshi and Tanji, 2000), which plays an essential role in higher-level cognitive processing, particularly the goal-processing operations (Fincham et al., 2002) and executing an action. In addition, previous reports also illustrated that the function of DLPFC including BA09 and BA46 is related to the executive function such as response control (Menon et al., 2001; Ridderinkhof et al., 2004). Importantly, during the stimuli period of spontaneous deceptive behavior, participants needed to make a decision on whether to tell truth or lie to the opponent, which might demand more cognitive efforts. By contrast, for the controlled behavior, participants were required to follow the instructions on the computer screen, which did not involve obvious cognitive efforts. Consequently, compared with that from the controlled behavior, enhanced brain power was discovered in the left frontopolar region and right DLPFC for the spontaneous behavior. Our results were also in line with previous findings that the functional brain networks of spontaneous deceptive behavior exhibited significant difference as compared to that from the controlled behavior (Zhang et al., 2016).

Meanwhile, the analysis results (Figure 6) of repeated-measures ANOVA are shown, which was performed to examine the brain power difference between the three conditions underlying the spontaneous deceptive behavior. Importantly, we discovered that there existed significant difference between the three cases during the performance of spontaneous behavior task. The brain regions that exhibited statistically significant power difference were the right frontopolar area (BA10) and the DLPFC (the left DLPFC, BA09, and BA46). Interestingly, previous fMRI studies also demonstrated that the PFC including the frontopolar area and DLPFC is the major cortical region within the “reward” neural network (Pochon et al., 2002; Haber and Knutson, 2010). Hence, the fMRI findings showed good agreement with our results regarding the activated brain regions. In addition, the results in Figure 6 demonstrated that there were statistical differences in the frontopolar area and the DLPFC between the case of truth-telling and lying. In the truth-telling cases (win–win, lose–lose), the participants’ answer were consistent with the real answer. However, for the lose–win case, participants needed to report the opposite of real answer to lie for receiving the reward. The frontopolar area and the DLPFC are parts of the anterior PFC and have been identified to play an important role in the processing of response control, which is discovered as the central of lying (Kozel et al., 2005; Priori et al., 2008; Mameli et al., 2010). It might be because of the differences that the participants choose to lie to get a reward or to present the fact as honest, which caused the significant differences in the frontopolar area and the DLPFC underlying the cases of using a truth-telling or a lying to deceive during the spontaneous behavior.

## Conclusion

To the best of our knowledge, it was the first time that the power analysis model, which combined the neuroimaging tool fNIRS and the Welch power spectrum estimation method, was utilized for the quantitative analysis of brain power in deception with different behavior states. We discovered that the demanding executive tasks under the spontaneous behavior produced significantly higher power than those under the controlled behavior in the PFC including the left frontopolar area and the right DLPFC. These findings showed that the power analysis method can provide us supplementary reference information to explore the neural mechanism of deception.

## Data Availability Statement

The original contributions presented in the study are included in the article/supplementary material, further inquiries can be directed to the corresponding author/s.

## Ethics Statement

The studies involving human participants were reviewed and approved by the Ethics Committees of the Zhejiang Normal University and the experimental tests were performed in accordance with the guidelines. The patients/participants provided their written informed consent to participate in this study.

## Author Contributions

JiaZ proposed the analysis method for processing fNIRS data. XL and LS designed the experiments. JiaZ and HR analyzed the data. XL prepared the Figures 1, 2B. HR prepared the Figures 3–6 and Table 1. XL and HR prepared the Table A1 and Figure 2A. JiaZ, HR, ZY, JinZ, XL, SM, and ZD wrote the manuscript. The others provided supports to this study during the experiments. All authors contributed to the article and approved the submitted version.

## Conflict of Interest

The authors declare that the research was conducted in the absence of any commercial or financial relationships that could be construed as a potential conflict of interest.

## Appendix

**TABLE A1:** The mean 3D MNI coordinates and associated brain regions for the 24 channels.

Channels	MNI coordinates (x, y, z)			Brodmann area	Probability of overlap
Ch01	−21	71	0	11—Orbitofrontal area	0.57
Ch02	−2	70	0	10—Frontopolar area	0.89
Ch03	21	72	−5	11—Orbitofrontal area	0.74
Ch04	−32	65	9	10—Frontopolar area	0.88
Ch05	−14	73	12	10—Frontopolar area	1
Ch06	12	73	12	10—Frontopolar area	1
Ch07	32	68	4	10—Frontopolar area	0.66
Ch08	−23	67	22	10—Frontopolar area	0.88
Ch09	−2	67	22	10—Frontopolar area	1
Ch10	20	70	19	10—Frontopolar area	1
Ch11	−32	55	30	46—Dorsolateral prefrontal cortex	0.97
Ch12	−13	62	35	9—Dorsolateral prefrontal cortex	0.50
Ch13	11	65	33	10—Frontopolar area	0.67
Ch14	31	58	28	46—Dorsolateral prefrontal cortex	0.77
Ch15	−20	52	42	9—Dorsolateral prefrontal cortex	0.93
Ch16	−4	56	43	9—Dorsolateral prefrontal cortex	0.95
Ch17	18	55	42	9—Dorsolateral prefrontal cortex	0.99
Ch18	−31	39	47	9—Dorsolateral prefrontal cortex	0.95
Ch19	−12	47	50	9—Dorsolateral prefrontal cortex	0.90
Ch20	10	49	51	9—Dorsolateral prefrontal cortex	0.84
Ch21	29	42	47	9—Dorsolateral prefrontal cortex	0.98
Ch22	−22	34	57	8—Includes Frontal eye fields	0.75
Ch23	−5	39	57	8—Includes Frontal eye fields	0.85
Ch24	18	37	58	8—Includes Frontal eye fields	0.76
